# Acupuncture in cancer care: recommendations for safe practice (peer-reviewed expert opinion)

**DOI:** 10.1007/s00520-024-08386-6

**Published:** 2024-03-14

**Authors:** Beverley de Valois, Teresa Young, Catherine Zollman, Ian Appleyard, Eran Ben-Arye, Mike Cummings, Ruth Green, Caroline Hoffman, Judith Lacey, Felicity Moir, Rachel Peckham, Jacqui Stringer, Susan Veleber, Matthew Weitzman, Kathrin Wode

**Affiliations:** 1 Supportive Oncology Research Team (SORT), East and North Hertfordshire NHS Trust Incorporating Mount Vernon Cancer Centre, Northwood, Middlesex UK; 2 Penny Brohn UK, Pill, Bristol, UK; 3 British Acupuncture Council, London, UK; 4 Clalit Health Services, Haifa, Israel; 5 British Medical Acupuncture Society, London Office, London, UK; 6 Imaging, Royal National Orthopaedic NHS Trust, Stanmore, Middlesex UK; 7 British Society for Integrative Oncology, London, UK; 8 Supportive Care and Integrative Oncology, Chris O’Brien Lifehouse Comprehensive Cancer Hospital, Sydney, NSW Australia; 9 University of Westminster (Retired), London, UK; 10 The Christie NHS Trust GB, Manchester, UK; 11 Integrative Medicine, Fred Hutchinson Cancer Center, Seattle, WA USA; 12 Integrative Medicine Service, Memorial Sloan Kettering Cancer Center, New York, NY USA; 13 Department of Neurobiology, Care Sciences and Society, Karolinska Institutet, Stockholm, Sweden

**Keywords:** Cancer, Acupuncture, Safe practice, Clinical recommendations, Integrative oncology, Supportive care

## Abstract

**Background:**

Up-to-date recommendations for the safe practice of acupuncture in integrative oncology are overdue with new cancer treatments and an increase in survivors with late effects of disease; 17 years have elapsed since Filshie and Hester’s 2006 guidelines. During 2022/2023 an expert panel assembled to produce updated recommendations aiming to facilitate safe and appropriate care by acupuncturists working with people with cancer.

**Methods:**

A core development team comprising three integrative oncology professionals comprehensively updated pre-existing unpublished recommendations. Twelve invited international experts (senior acupuncturists with and without experience of working in oncology settings, oncologists, physicians and nurses trained in integrative oncology, researchers, academics, and professional body representatives) reviewed the recommendations. In multiple iterations, the core team harmonised comments for final ratification. To aid dissemination and uptake the panel represents national and international integrative oncology associations and major cancer treatment centres in Europe, USA, Australia, and the Middle East.

**Results:**

These recommendations facilitate safe care by articulating contra-indications, cautions, and risks for patients both on and off treatment (surgery, SACT, radiotherapy). Situations where acupuncture may be contra-indicated or practices need adapting are identified. “Red and Amber Flags” highlight where urgent referral is essential.

**Conclusion:**

These are the first international, multidisciplinary peer-reviewed recommendations for safe acupuncture practice in integrative oncology. Concerns about safety remain a significant barrier to appropriate referral from oncology teams, to use by acupuncturists and to uptake by patients. Disseminating trustworthy, widely accessible guidance should facilitate informed, confident practice of acupuncture in and outside of oncology healthcare settings.

## Purpose

Acupuncture can be a helpful intervention in the multidisciplinary care of people living with and beyond cancer. It can be beneficial in relieving unpleasant side effects of cancer treatments, in many cases offering a non-pharmaceutical option that can be used during ongoing treatment. It can also help people recover after they have completed treatment or be used to manage issues during late stage and end of life. It can be administered as a person-centred intervention, individualised to take into account a person’s preferences, needs, and values, which can guide clinical decision making [1]. Safe compared to most conventional treatment, there are special considerations for the safe practice of acupuncture in the cancer population.

The purpose of these recommendations is to facilitate safe clinical practice and they aim to:

- Support, inform, and empower acupuncturists who are treating people with a cancer diagnosis, and enable them to offer acupuncture as a safe, appropriate adjunct to routine treatment and care.
- Ensure that practitioners of acupuncture are aware of the contra-indications, cautions, and risks of using acupuncture in this population.

They are intended for use by any practitioner of acupuncture using any form of acupuncture and working in any setting, including in:

- An integrative oncology setting, complying with established policies, and possibly with access to patients’ medical records (including data regarding blood counts)
- A not-for-profit cancer support charity setting
- A non-oncological healthcare setting (e.g. physiotherapy unit or GP practice)
- A hospice setting
- The community, independently, outside of a medical setting (e.g. private acupuncture practice).

These recommendations may also be used to inform oncology healthcare professionals (who are not acupuncturists) and people with cancer about the safe practice of acupuncture. They may also inform policies and protocols in oncology settings.

## How to use this document

This document covers a wide range of acupuncture practitioners working in diverse settings; therefore, some acupuncturists will have access to patients’ medical information whilst others may not. These guidelines should be used in conjunction with local policies and procedures, where these apply.

Acupuncturists working without access to patient medical records may not have data for the recommended cut-off points, for example blood counts for assessing neutropenia and thrombocytopenia. If they are treating patients who are receiving concurrent systemic anti-cancer treatments (SACT) such as chemotherapy, they should ideally be in communication with the patient’s healthcare team to establish recommended procedures for that patient. If this is not possible, the acupuncturist should be experienced in treating people with cancer and have a good working knowledge of the patient’s cancer type and treatment protocol including natural history of the disease and potential complications.

All acupuncturists treating people with cancer, at any stage of their illness, should be aware of Red and Amber Flags associated with the disease and its treatments (see Recognising potential "Red and Amber Flag" symptoms needing referral for medical assessment/management) and precautions for vulnerable patients. They should also know where and when to refer and when not to treat.

## Definitions and scope

“Acupuncture” refers primarily to the insertion of fine solid needles under the skin. Although this is the main method used by acupuncturists, many practitioners also use associated modalities including electroacupuncture, in-dwelling needles, moxibustion (or moxa, a thermal treatment using the smouldering herb *artemisia vulgaris* (also known as *artemisia argyi*)), acupressure, ear seeds, cupping, and lifestyle advice.

Chinese herbal medicine, which is practised by many acupuncturists, is not within the scope of these guidelines.

**Fig. 1:**
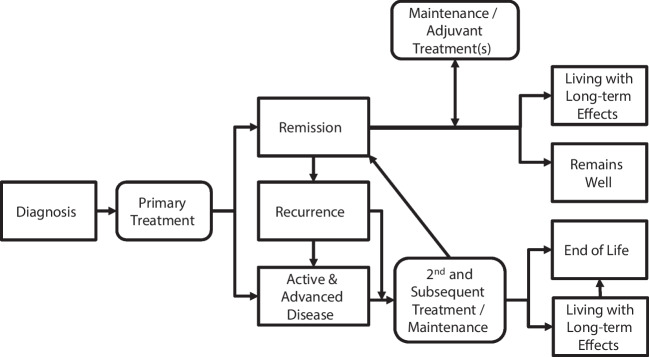
The cancer survivorship pathway

These recommendations are intended to apply to people with a cancer diagnosis at any stage of the cancer survivorship pathway. A simplified diagram showing key stages is presented in Fig. 1. This pathway describes the different phases of health or illness a person may experience from the point of cancer diagnosis onwards. It was intended to help clarify thinking about the support individuals might need at different times after cancer diagnosis [2].

## Background

Acupuncture is widely used for people with a cancer diagnosis to relieve symptoms related to cancer and cancer treatments. Several guidelines exist (Table 1) recommending acupuncture for symptoms such as fatigue [3, 4], pain (myofascial, arthralgic, musculoskeletal) [5–7], chemotherapy-induced peripheral neuropathy (CIPN) [5, 7], chemotherapy-induced [4] and anticipatory nausea and vomiting [8], radiotherapy-induced xerostomia (RIX) [9], and anxiety [10]. Clinically, acupuncture is used to manage many additional symptoms, such as depressive symptoms and hot flushes [4, 11].

**Table 1:** Examples of guidelines recommending acupuncture for people with a diagnosis of cancer

Guideline	Publication date	Cancer type/population	Symptoms covered include	Hyperlink
Integrative Oncology Care of Symptoms of Anxiety and Depression in Adults With Cancer: Society for Integrative Oncology-ASCO Guideline	2023	Adults with cancer, either during cancer treatment or post-treatment	Anxiety, depression	https://ascopubs.org/doi/pdf/10.1200/JCO.23.00857?role=tab
Integrative Medicine for Pain Management in Oncology: Society for Integrative Oncology-ASCO Guideline	2022	Patients of any age diagnosed with any cancer who are experiencing pain during any stage of their cancer care trajectory	Pain including aromatase inhibitor-related joint pain (arthralgia); general pain or musculoskeletal pain from cancer; chemotherapy-induced peripheral neuropathy (CIPN); pain associated with cancer treatments	https://ascopubs.org/doi/full/10.1200/JCO.22.01357
Clinical practice guidelines on the evidence-based use of integrative therapies during and after breast cancer treatment	2017	Breast cancer	Anxiety, chemotherapy-induced nausea and vomiting (CINV); depression/mood disturbance, fatigue, pain, quality of life, vasomotor symptoms	https://acsjournals.onlinelibrary.wiley.com/doi/epdf/10.3322/caac.21397
Complementary therapies and integrative medicine in lung cancer: Diagnosis and management of lung cancer, 3rd ed: American College of Chest Physicians evidence-based clinical practice guidelines	2013	Lung cancer	Nausea and vomiting from either chemotherapy or radiation therapy; cancer-related pain and peripheral neuropathy	https://journal.chestnet.org/action/showPdf?pii=S0012-3692%2813%2960303-7
NCCN Guidelines® Survivorship	2023	Survivorship, all cancers	Fatigue, vasomotor symptoms (hot flushes, night sweats), arthralgias, myalgias, myofascial, neuropathic pain in survivors	https://www.nccn.org/professionals/physician_gls/pdf/survivorship.pdf
NCCN Guidelines® Cancer-Related Fatigue	2023	Cancer-related fatigue in children/adolescents and adults	Cancer-related fatigue	https://www.nccn.org/professionals/physician_gls/pdf/fatigue.pdf
NCCN Guidelines® Antiemesis	2023	Patients receiving anti-cancer agents and/or radiotherapy	Anticipatory nausea and vomiting	https://www.nccn.org/professionals/physician_gls/pdf/antiemesis.pdf
NCCN Guidelines® Palliative Care	2023	To anticipate, prevent, and reduce suffering; promote adaptive coping; and support the best possible quality of life for patients/families/caregivers, regardless of the stage of the disease or the need for other therapies	Pain, nausea, and vomiting (chemotherapy induced and non-specific)	https://www.nccn.org/professionals/physician_gls/pdf/palliative.pdf

To access NCCN Guidelines, it is first necessary to create an account. The links direct to the account log-in page in the first instance

*ASCO* American Society of Clinical Oncology; *NCCN* National Comprehensive Cancer Network

Acupuncture also addresses symptom clusters, rather than single symptoms [11, 12]. This, in addition to its capacity to span physical, emotional, psychological, and even spiritual concerns, makes it a particularly useful treatment option in the practice of oncology. Day-to-day clinical experience as well as patient feedback confirm that acupuncture is helpful to address many of the troublesome aspects of the cancer experience (Table 2), from addressing complex presentations of acute and chronic physical symptoms and treatment side effects, to alleviating psychosocial consequences, to helping people cope better [13] and improve resilience [14] and quality of life [4] after a cancer diagnosis.

**Table 2:** Cancer treatments and some consequences of cancer and its treatments

Cancer treatments include:	
• Surgery • Systemic anti-cancer treatments (SACT) (chemotherapy, immunotherapy, targeted biological therapies) • Radiotherapy • Hormonal treatments.^*^	
Some physical consequences of cancer and cancer treatments (listed alphabetically)	Some psycho-socio-spiritual consequences of cancer and cancer treatments (listed alphabetically)
• Blood cell count abnormalities including anaemia (reduced haemoglobin or red blood cells), neutropenia (reduced white blood cells), and thrombocytopenia (reduced platelets) • Bowel or bladder dysfunction • Chemotherapy-induced pain, altered sensation, and/or numbness in the feet or hands (CIPN — chemotherapy-induced peripheral neuropathy) • Digestive issues • Dry mouth • Physical fatigue • Hair loss • Hormonal side effects (e.g. flushing, sweats, arthralgia, loss of libido) • Lymphoedema • Nail changes (nail dystrophy) • Nausea and vomiting • Pain (including chronic pain) • Difficulties relaxing • Skin changes • Sleep problems • Taste disturbance • Weight loss/gain	• Anxiety • Body image concerns • Cognitive/emotional/social fatigue • Depression • End-of-life anxiety • Fear of recurrence • Financial pressures • Hopelessness • Inability to plan for the future • Loss of: • Confidence • Control • Meaning and purpose • Motivation • Role/vocation • Psychosexual issues • Relationship changes

^*^Hormonal treatments are technically classified as SACTs. We discuss them separately as their consequences are generally less severe than those related to chemotherapy, etc., and they are prescribed for long-term use (often for many years)

## Methods

Guidance published by Filshie and Hester in 2006 [15] was updated by Zollman in 2018 for in-house use by a non-profit organisation (Penny Brohn UK). The organisation offered complementary therapy as an integrative adjunct to clinical cancer treatment and care and the guidance was intended to ensure safe practice. It also aimed to reassure individual cancer clinicians and organisations with less experience of Complementary Traditional and Integrative Medicine (CTIM) therapies that acupuncture was being given appropriately and with due evidence-informed consideration of important potential contra-indications and clinical cautions.

In 2022, a core development team (B. d. V., T. Y., C. Z.) comprehensively updated the unpublished 2018 document. This was peer-reviewed by 12 of 16 invited international experts, comprising senior acupuncturists with and without experience of working in oncology settings, oncologists, physicians and nurses trained in integrative oncology, researchers, academics, and professional body representatives. Experts’ comments were then harmonised by the core team. After two iterations of this process, the document was issued for ratification by the expert panel and finalised.

To aid dissemination and uptake of the recommendations the expert panel represents national and international integrative oncology associations and major cancer treatment centres in Europe, USA, Australia, and the Middle East.

## Acupuncture in integrative oncology — fundamental requirements

### Registration, insurance, and scope of practice

Acupuncturists treating people who have been diagnosed with cancer should be registered with a recognised professional body and fully insured. These guidelines should be read in conjunction with the:

- Code of practice and ethics of the relevant recognised professional body for acupuncture.
- Local policies for the provision of acupuncture within a cancer service (where applicable).

### Working as part of a multi-disciplinary team

Acupuncturists working with people with cancer in a healthcare environment should ideally work as part of the multi-disciplinary oncology team. Whatever their working environment, the acupuncturist should:

- Refrain from making any claims that they can “treat” or cure cancer (in the UK, this is prohibited by law under the Cancer Act 1939) and must be realistic and truthful about the aims and likely effects of acupuncture.
- Communicate any new, unexplained symptom or sign to the clinical team, and/or refer the patient back to the appropriate healthcare professional in a timely manner.
- Refrain from expressing any opinions about, or suggesting any changes to, medically prescribed medications or treatments (e.g. if a patient is experiencing side effects which are not adequately controlled) and should encourage the patient to discuss concerns with their clinical team.
- Follow their professional body’s and/or local guidance, whichever is the more appropriate, regarding infection prevention and control. Special measures such as those related to COVID-19 are likely to be critically important when supporting those who have had a cancer diagnosis.

### Consent

Good practice includes obtaining consent for acupuncture treatment, for the protection of both patient and practitioner. Consent is the voluntary agreement given by a person to allow something to happen to them, and/or to be done to them, and/or to allow their participation in something.

It is fundamental that every adult with capacity has the absolute right to determine what happens to their own body. This right is protected by law. Valid consent must meet the following:

- The patient must have the capacity to give their consent
- The consent must be given voluntarily
- The patient must have been given adequate information and an opportunity to ask questions to enable them to make an informed decision [16].

If any one of the requirements outlined are not met, then the consent may not be legally valid.

Cancer patients are often vulnerable physically and/or psychologically. If a patient is deemed to be vulnerable but nonetheless has capacity, particular care must be taken to ensure that consent to acupuncture is given voluntarily and without any duress. Otherwise, someone with a Lasting Power of Attorney (or equivalent) may be able to give consent on the patient’s behalf.

How consent is documented will be influenced by the setting where acupuncture is being given and should be in accordance with appropriate guidelines of the institution or the relevant professional body.

### Treatment records

The acupuncturist must keep adequate concurrent records of every treatment encounter, including records of any adverse events, however minor.

## Acupuncture in the context of integrative oncology

Acupuncture practice can easily be adapted to the cancer context, and all the usual guidelines for safe practice should be observed when treating patients living with and beyond cancer. To adapt it to suit patients with serious illness, an appropriate assessment of the patient’s situation is needed. If the patient is very unwell, very frail, very elderly, very young, or is uncertain about the use of needles (many patients with cancer develop a degree of needle phobia after repeated blood tests and intravenous treatments), modifications may be necessary (discussed below). Acupuncture treatment should always be tailored to the needs of the patient, and the contraindications for treatment (Table 3) observed.

**Table 3:** Contraindications

Acupuncture should be avoided if:
• The patient does not want a treatment. • No appropriate consent for treatment is in place. • The consultant responsible for the patient has withheld consent for acupuncture for any reason. • The clinical team responsible for the patient have communicated any significant concerns about acupuncture to the patient which have not been addressed. • The patient is profoundly immuno-suppressed and/or in isolation (e.g. post-bone-marrow transplant) unless acupuncture is authorised by the medical team. • The patient has had recent radioactive oral or intravenous treatments (e.g. theranostics or radioiodine) or investigations involving radioactive materials (e.g. some types of nuclear medicine scans) and is still within the period when it is deemed unsafe for other people to have close contact with the patient. Whether this is an issue will be dictated by the length of any post-radiation restrictions and these will have been clearly communicated to the patient, if relevant. • The patient has a febrile illness, or any cellulitis or local infection in the area where the acupuncturist intends to place needles, and there are no alternatives to needling there. • The acupuncturist has an infectious illness or has been in close contact with someone with a serious infectious illness, as this could be passed to an immuno-compromised patient with serious consequences.

When taking a whole person approach (considering the wellbeing of the whole person, not just the illness) the acupuncturist can always offer something, even if acupuncture needling itself is deemed not to be appropriate for any reason (Table 3). Options (see Related Techniques) may include a supportive listening ear, demonstrating how to use seeds or beads, acupressure points or moxibustion for self-use at home, or signposting to other suitable interventions (e.g. shiatsu, acupressure, reflexology). It is as important to know when not to treat with acupuncture, as it is to know when and how to treat.

Acupuncture is a very flexible modality, and acupuncturists should be conversant and comfortable with adapting approaches in circumstances where needling should be avoided (e.g. avoiding needling an at-risk area of the body such as a lymphoedematous limb), or when acupuncture is contraindicated. Amongst the many possible alternative approaches, some to consider include using distal rather than local points, needling the contralateral side, and using microsystems such as ear acupuncture.

### Infection prevention and control

Clean needle technique should be practiced. This encompasses using disposable single-use needles and immediately discarding them into a sharp container which is disposed of as per local guidance.

Swabbing the local area with alcohol or anti-septic prior to needling may be required by local policies.

All acupuncturists should be scrupulous with infection control measures. Local policies for hand hygiene and for wearing of personal protective equipment (PPE) should be observed. Those treating neutropenic patients, or patients at risk of neutropenia, should be extra vigilant and should avoid giving treatments or any form of face-to-face consultation if they:

- Feel they are at any risk of passing on an infectious disease such as an upper respiratory tract infection or infectious gastroenteritis.
- Have been in contact with someone in, or suspected of being in, the contagious phase of a more significant infectious disease such as chicken pox, shingles, measles, and COVID.

### Considerations for needling and techniques related to acupuncture

- With fragile or vulnerable patients, acupuncturists may consider adopting gentle needling techniques, including mild (if any) manual stimulation using thin needles [17].
- Needling into breast tissue is contraindicated by some schools of acupuncture and regulatory bodies, although we have been unable to find any rationale or evidence for or against for this advice.
- Indwelling acupuncture needles pose a slight increased risk of infection and should be avoided where there is any risk of reduced immune function. They should also be avoided in patients with:
  - Valvular heart disease
  - Bloodborne infections (e.g. hepatitis B or C, HIV) where a needle which falls out or becomes displaced could pose a risk to others
  - A tendency to keloid scar formation.
- When using non-penetrative seeds or beads for ongoing stimulation, they should be applied to unbroken skin after checking for allergies to adhesive tape.
- Both indwelling needles and metal beads should be removed prior to imaging.

### Related techniques

Depending on the clinical setting where acupuncture is being offered, and the expertise and training of the acupuncturist, other non-penetrative modalities may be considered in addition, or as alternatives, to needling. This may be especially relevant for patients undergoing active treatment. Examples include:

- Acupressure (recommended for CINV, used in clinical setting for pain, stress management, and fatigue [4])
- Cupping
- Moxibustion
- Heat lamps
- Non-penetrative beads or seeds.

Care should be exercised when using moxa and heat lamps as chemotherapy and radiotherapy can increase skin sensitivity and the propensity to burn. Neuropathy may reduce sensitivity to heat, with potential for injury. Moxa therapy is often limited in oncology settings due to smoke restrictions and should be avoided in hospital and clinical areas where flammable gases are present.

When using modalities involving deep or intense pressure (acupressure, cupping), the advice for all touch therapies should be followed, i.e.:

“The application of deep or intense pressure is not recommended near cancer lesions, enlarged lymph nodes, radiation field sites, medical devices (such as indwelling intravenous catheters or stoma bags), or anatomic distortions or in patients with a bleeding tendency” [9]. (“Anatomic distortions” refers to areas that are not in their usual shape or position, and may be the result of surgical interventions.)

Firm or focussed pressure on limbs with or at risk of lymphoedema should be avoided [18] (see the “Lymphoedema and risk of lymphoedema” section).

## Potential adverse events associated with acupuncture in cancer care

### General considerations

Numerous studies report that acupuncture is a safe intervention when performed by qualified practitioners, and in the general population adverse events are rare [19–23]. The most commonly reported minor adverse events of acupuncture treatment are transient bleeding and pain at the needle site, occurring in less than two in 1000 treatments [9]. Mild bruising, drowsiness, headache, and local skin irritation may also occur, as well as dizziness and fainting (vasovagal reaction) in about 1% of treatments [24]. Pneumothorax, although rare, is a concern (see the “Pneumothorax” section).

In the only study of safety of acupuncture in oncology to date the authors conclude that acupuncture is as safe as sham acupuncture and active controls in oncological patients, although better and more consistent reporting of adverse events in research studies is required [25].

People undergoing active treatment for cancer may be less resilient and therefore more susceptible to adverse events. All acupuncturists should be conversant with the Red and Amber Flag symptoms related to cancer and cancer treatment (Table 4).

**Table 4:** Red and Amber Flag symptoms related to cancer or cancer treatments

**General note:** These recommendations should be considered in relation to any local guidance and procedures, which take precedence.	
**RED FLAG: Refer urgently for medical assessment. Do not treat with acupuncture.**	
**Recommended actions for any of the symptoms/situations are:** • Urgent referral (following any local procedures) for assessment/medical review in a relevant facility, e.g., Acute Oncology Services (AOS) in the UK or hospital Accident and Emergency (A&E) department. • Following medical assessment, and if Red Flag symptom(s) are ongoing, give acupuncture only with medical consent/supervision.	
**Anorexia (severe)/weight loss/malnutrition:** No/minimal oral intake or urinary output for > 12 hours. Decreased oral intake in association with significant unintentional weight loss (> 10% loss in previous 3-6 months) or malnutrition (MUST score of ≥ 2 [28]).	
**Auto-immune disease – NEW if on immunotherapy** See Table 5 below and the section “Auto-immune reactions to immunotherapy” below for potential reactions.	
**Bleeding and bruising:** Unexpected, severe, or massive bleeding or widespread spontaneous bruising (including widespread purple petechial rash, haemoptysis, haematemesis, significant nosebleed).	
**Cognitive:** New or worsening confusion or disorientation/ cognitive disturbance/reduction in conscious level.	
**Constipation: Severe** No bowel movement for 72 hours over usual regularity pre-cancer treatment.	
**Diarrhoea:** Diarrhoea sufficient to cause dehydration or metabolic upset, bleeding, or significant pain e.g., > 7–9 episodes per day; or severe increase in ostomy output; or incontinence, severe cramping, or bloody diarrhoea; also associated exhaustion, dehydration, feeling unwell.	
**Dizziness:** New or significant dizziness/light-headedness/postural hypotension.^1^	
**Dyspnoea/shortness of breath - new:** If newly short of breath with minimal exertion, or at rest.	
**Fatigue:** New severe fatigue or sudden significant increase in fatigue (as per “General condition deterioration” below) that is not already being monitored/managed by the medical team.	
**Fever:** • If ≥ 37.5^◦^, or < 36^◦^ or generally unwell, and/or has received active anti-cancer treatment within the previous 6 weeks or if known to be at risk of prolonged immune compromise (e.g., some haematology patients, or patients with bone marrow involvement). • If > 38.0^◦^ but not receiving active anti-cancer treatment or not at risk of immunosuppression.	
**General condition deterioration (performance status)/generally unwell:** Sudden, significant deterioration in general condition – e.g., generalised weakness, significant unintentional weight loss, fatigue, worsening mental state, reduced ability to mobilise or self-care that is not already being monitored/managed by the medical team.	
**Infection:** If receiving or has received potentially immunosuppressive anti-cancer treatment within the previous 6–8 weeks or if immunocompromised AND experiencing any symptoms of infection and feeling generally unwell, even without fever; including any shivering, chills or shaking episodes; cough; pain, burning/stinging or difficulty passing urine.	
**Nausea, vomiting:** Nausea or vomiting sufficient to cause dehydration/ketosis/reduction in urinary output, if no significant intake of food or fluids, or > 6–10 episodes of vomiting in previous 24 hours; also associated exhaustion, dehydration, feeling unwell.	
**Neurological symptoms – new:** Any new persistent neurological symptoms, e.g., change in vision, hearing or conscious level; severe headache; numbness, tingling, reduced sensation; weakness; impairment of bowel or bladder function.^2^	
**Oral stomatitis (severe inflammation or ulceration of the lining of the mouth):** Oral stomatitis with painful mouth and difficulty eating and drinking sufficient to limit oral intake of fluids and food.	
**Pain:** Severe, uncontrolled pain interfering with daily activities.	
**Rash:** Any petechial rash. Any new rash covering > 10% of the body surface area with or without symptoms such as pruritus, burning or tightness.	
**Swelling, redness or tenderness:** Swelling, redness or tenderness in a limb or localised area of the head, neck, or torso, especially if there is an indwelling intravenous catheter present.^3^	
**Urinary disorder:** Severe symptoms; possible obstruction/retention; new incontinence; new or increasing haematuria; significant reduction of output.	
**AMBER FLAG: Acupuncture can be given but prompt communication with medical team is needed to ensure they are aware.**	
**Recommended actions for any of the symptoms/situations are:** • PROMPT communication to the patient’s own oncology or medical team for further assessment if there is NEW onset or significant worsening of any of the following. • URGENT medical assessment if two or more Amber Flags present simultaneously and the patient is generally unwell (i.e., consider as a Red Flag and refer urgently as above) • Following medical assessment, give ongoing acupuncture ideally with medical team’s knowledge and agreement.	
**Anorexia:** Significantly reduced eating and drinking with > 5% unintentional weight loss (or a newly increased MUST score of 1) but a normal urinary output [28]. If urine output is significantly reduced treat as Red Flag as above.	
**Bruising:** Multiple sites of bruising or one large site.	
**Constipation:** Moderate – no bowel movement for 48 hours over usual regularity pre-cancer treatment.	
**Diarrhoea:** Increase of 2–6 bowel movements a day over pre-treatment frequency, nocturnal movements, or moderate cramping.	
**Fatigue:** If moderate but affecting daily function.	
**Lymphoedema (symptomatic):** Swelling of a limb or body part that is not red or tender but is causing discomfort.	
**Nausea, vomiting:** Mild – moderate nausea or vomiting that is not adequately controlled with current anti-sickness medication (medical team may be able to prescribe more effective medication).	
**Neurological:** Mild or moderate sensory loss, moderate paraesthesia (notify chemotherapy staff if likely to be peripheral neuropathy caused by ongoing SACT), mild weakness with no loss of function, visual or other sensory disturbance.	
**Pain:** **New unexplained significant or persistent pain. Any new pain should be investigated promptly**. **Any severe or uncontrolled pain should be treated as a Red Flag symptom.** If pain associated with redness or swelling, consider thrombosis or cellulitis (see below). If back pain, check for signs of neurosensory or motor loss.^4^	
**Rash:** Rash covering < 10% of body surface area that is limiting normal activities of daily living	

^1^In a patient who has received immunotherapy underlying cause may be Auto-Immune Addison’s Disease (hypoadrenalism)

^2^Consider possible spinal cord compression as prompt treatment can prevent permanent paralysis

^3^Consider possible thrombosis or cellulitis

^4^May indicate spinal cord compression (SCC)

Acupuncturists working in healthcare settings should also be familiar with any:

- Local risk assessments for acupuncture and seek to reduce the risk of adverse events.
- Local policies and procedures on who to contact should adverse events occur.

Acupuncturists working independently should contact the patient’s doctor in the case of any significant medical adverse event from acupuncture or call an ambulance if there is any immediate danger.

### Bleeding and bruising

Some people with cancer are at risk of increased bleeding either because of treatment-related side effects or because of cancer deposits causing bone marrow or liver dysfunction. They may have a reduced platelet count (see the “Thrombocytopenia” section) or impaired clotting factors. Some patients with cancer will be on medication to treat or prevent venous thrombosis (see the “Anti-coagulant therapies” section). The acupuncturist should be aware of the individual patient’s risk factors.

Even in the cancer population, the risk of significant bleeding following acupuncture is extremely low, but patients should be warned about the potential increased risk of bruising and minor bleeding [26]. Acupuncture should be avoided if there is evidence of spontaneous bleeding, bruising, or haematoma. Vigorous needling techniques, particularly into enclosed fascial compartments of the leg or joint spaces, should be avoided if there is an increased risk of bleeding.

### Needle shock and fainting (vasovagal reaction)

Cancer patients may have an increased risk of vasovagal attacks (fainting) because of their cancer diagnosis, their treatment, or because of anxiety about needles. Patients undergoing active cancer treatment may be fatigued, exhausted, or may not have eaten recently. Where possible, the first acupuncture treatment should not be given on an empty stomach and ideally be given with the patient lying down. Patients should be monitored closely, especially during their first treatment.

Care should be taken when offering acupuncture to people with a history of fainting during venepuncture (taking blood), having an intravenous catheter inserted, or during previous acupuncture. If it is jointly agreed that the potential benefits outweigh the risks, the patient should be treated lying down, in a position where they cannot fall off the bed/couch.

### Pneumothorax

Pneumothorax, although rare, is a concern in all populations. A Taiwanese study of the general population reported an incidence of 1.75 per million from 191,745 patients receiving 2,684,774 acupuncture treatments over at-risk areas (thorax or neck). Highest risk was associated with the patient being male, having a history of lung disease (including lung cancer), or having thoracic surgery [27]. Acupuncturists should observe usual precautions to minimise the risk of pneumothorax, paying special attention to those with low Body Mass Index, i.e. BMI < 15 (see the “Cachexia/anorexia/low BMI” section).

## Cancer-specific considerations

### Understanding oncology

Cancer is not a single diagnosis and even within a single tumour site, histological variants affect potential treatments, prognosis, and outcomes. All practitioners of acupuncture who accept clients who have had a cancer diagnosis should have a good background knowledge of the client’s particular cancer, including the natural history of the disease and cancer treatments and their related consequences. This applies equally to practitioners who are medically trained and to those who are not. Ideally, practitioners of acupuncture working with patients receiving active treatment, particularly SACT, or advanced palliative care should undertake some training in integrative oncology, preferably within an oncology centre offering integrative approaches. Where this is not possible, all practitioners should make sure they are aware of and confident in recognising “Red and Amber Flag symptoms” which require further medical assessment or treatment (Table 4) and that they are fully conversant with the guidance presented here.

### Recognising potential “Red and Amber Flag” symptoms needing referral for medical assessment/management

Patients currently receiving active anti-cancer treatments should have received instructions for what to do in Red Flag situations (see Table 4), for example calling the local Acute Oncology Service’s (AOS) emergency hotline. Acupuncturists who become aware of any “Red Flag symptoms” should advise patients on treatment to follow the instructions they have been given by their oncology team. However, if their condition is immediately life-threatening, they will need urgent transportation to an emergency care (A&E) department. Acupuncturists should encourage patients to report any new persistent symptoms to their oncology teams.

## Modifying acupuncture approaches for specific cancer-related conditions (listed alphabetically)

### General recommendation for new unexplained pain or other new symptoms

Severe uncontrolled pain is a medical emergency which should be addressed urgently. New pain or other symptoms (e.g. headache, unexplained nausea, or digestive symptoms) reported by cancer survivors should be investigated thoroughly, by full medical (and radiological, if appropriate) assessment. Care must be taken to ensure they are not a sign of disease progression, recurrence, or onset of second primary cancers, even if acupuncture improves the symptoms [5, 29, 30].

Once appropriate medical assessment/management is in place and with medical agreement, acupuncture may provide a useful adjunctive symptom management strategy which may help reduce the need, dose, and side effects of analgesic and other medication [7].

### Anti-coagulant therapies

Reports of serious adverse effects from acupuncture in stable anti-coagulated patients are extremely rare [26]. Minor adverse effects which may be more common in anti-coagulated patients include minor bruising and mild, superficial bleeding. Most anti-coagulants (including injectable low molecular weight heparin and direct oral anti-coagulants (DOACs)) reach their therapeutic dose immediately but achieving the correct dose of Warfarin often takes more time. Acupuncture should be used with caution if the patient is being anti-coagulated and there are concerns that their anti-coagulation may be unstable, i.e. if the patient has recently been started on Warfarin and they are still in the balancing phase, or their dose has been changed recently, as they may be either under- or over-anti-coagulated. Once the Warfarin dosage is stable and time for blood clotting (as measured by the International Normalised Ratio (INR)) is in the desired range, then acupuncture can be provided with appropriate discussion with the patient of the slightly increased risk of minor bruising or bleeding.

### Cachexia/anorexia/low BMI

People with profound cachexia or anorexia and a very low BMI (BMI < 15) have reduced subcutaneous fat and muscle and are theoretically more at risk of needles penetrating an underlying organ, when needling over the front or back of the chest or abdomen. For these patients, superficial rather than deep needling is indicated if using points on the upper torso, including chest, upper trapezius, upper back [31], and abdomen. Choosing distal points may be a preferable option.

### Compromised immune function

It is important to remember that most cancer survivors who have completed treatment, and many who are on longer term non-immunosuppressive treatments (e.g. hormonal therapy), have normal immune function. However, some may be at risk of neutropenia (low white blood cell count), either because of cancer treatment-related side effects or bone marrow infiltration by cancer (see the “Neutropenia” section).

### Deep vein thrombosis

People with cancer are at an increased risk of deep vein thrombosis (DVT), which is a Red Flag (see Table 4). Acupuncture should not be given to a limb where there is, or has been, a confirmed or potential DVT (asymmetrically swollen, red, hot limb) until after the resolution of the DVT symptoms.

When a patient is having anti-coagulant (blood thinning) as treatment or secondary prevention after a suspected or confirmed DVT, they should be treated as per the guidance for “Anti-coagulant therapies.”

### Infection or open wounds

Patients with cancer may be more susceptible to infections of all types. Acupuncture in the local area should be avoided when the patient presents with:

- Cellulitis, an infection of the skin and subcutaneous tissue characterised by local redness, and sometimes with “shininess” of the skin, warmth, pain, and swelling (untreated cellulitis is a Red Flag — see Table 4).
- An open wound.

If the patient does not have fever and is well in themselves, acupuncture can be provided elsewhere on the body.

### Lymphoedema and risk of lymphoedema

Lymphoedema results from a failure of the lymphatic system [32]. Consequences are swelling, skin and tissue changes, and a predisposition to infection. The condition requires specialist care to prevent progression. Risk factors for lymphoedema include surgery and radiotherapy when they involve the regional lymph nodes. The risk of lymphoedema and infection (cellulitis) can remain for many years after completion of cancer treatment and is probably best regarded as life-long [33].

Whilst lymphoedema experts acknowledge the lack of robust evidence for guidelines for reducing risk of developing lymphoedema [34, 35], current lymphology expert opinion advises that needle punctures (e.g. blood drawing, vaccination, intravenous cannulation) should not be allowed in an area with or at-risk of lymphoedema, unless there are life-compromising and life-saving situations in which intravenous or surgical interventions on the at-risk or affected area are unavoidable [18]. This advice encompasses tattoos and acupuncture as well as interventions that put pressure on a limb such as blood pressure cuffs, tourniquets, and other forms of firm pressure.

Many patients with or at risk of lymphoedema find having acupuncture is beneficial. In the absence of clear evidence as to the benefit or harm of applying acupuncture to an affected limb, best practice would advise caution and avoid needling limbs with or at-risk of lymphoedema [9, 15, 18].

### Tumours and metastases

Direct needling into acupuncture points in or in the immediate vicinity of tumours should be avoided. Use distal points in patients with locally advanced malignant lesions in preference to penetration or proximity to the tumour itself to reduce any local disruption of tumour. Similarly, in the absence of clear evidence to the contrary, needling in the sites near or over metastases should be avoided [36].

### Unstable spine

Local paraspinal acupuncture for back pain in patients with known metastatic or spinal disease should only be performed after consultation with the oncology/palliative care team. Due to a potential (anecdotal) risk that acupuncture may relax paraspinal muscles which are helping to stabilise an otherwise unstable spine, patients should be warned to report any new or changed neurological symptoms (numbness, tingling, weakness, changes in bowel or bladder function) to their medical team immediately as a Red Flag (see Table 4).

## Acupuncture during and after specific cancer treatments

### Surgery

If the patient has had recent surgery, acupuncture needling to the local area should be avoided until stitches have dissolved or been removed and any local swelling or erythema (redness) has settled. In the case of laparoscopic (keyhole) surgery, the internal area affected by surgery will be more extensive than any external scarring so the whole area (e.g. abdomen or pelvis) should be avoided in the immediate recovery phase. The duration of recovery depends on the procedure performed. Patients will generally be advised when they are fit to drive again, and this is a good indication of when tissue repair will be sufficient for local superficial needling to the area. Any post-operative local infection should be treated before acupuncture is given.

#### Implants, prostheses, and other medical devices

Due to the increased risk of introducing infection in a site where there is foreign material in the body, avoid direct needling into, or close to:

- Prostheses, implants, ports, tissue expanders, pacemakers etc.
- Insertion sites of recent or current IV cannulae or lines.

#### Craniotomy and cranial defects

Needles should not be inserted over a craniotomy flap where a portion of the skull has been surgically removed and the brain is covered by skin and soft tissue only, as the natural barriers to infection are reduced and there is an increased risk of local skin infection causing meningitis or encephalitis.

### Systemic anti-cancer therapies

#### Neutropenia

Most forms of chemotherapy cause at least temporary periods of neutropenia (low white blood cell count) during active treatment. The lowest count reached is called the nadir. The severity and duration of the neutropenia depends on the type of chemotherapy and the patient’s background disease and condition. Neutropenia usually resolves within 6 weeks after completion of chemotherapy treatment but may be more prolonged.

Targeted treatment and immunotherapy usually cause a less profound and significant neutropenia but, in some cases, low white blood cell counts remain a problem. Local radiotherapy rarely causes neutropenia but Whole-Body Radiotherapy (e.g. in preparation for a bone marrow transplant) causes a severe neutropenia which is usually managed in hospital. Patients are usually made aware if their white blood cell counts are lower than normal.

Neutropenia increases the risk of overwhelming infection (neutropenic sepsis) as the body lacks an effective immune response to pathogens. Sepsis is a medical emergency and requires immediate hospital admission and treatment with intravenous antibiotics. There is therefore understandable concern about avoiding any procedures which may increase the risk of introducing infection into the bloodstream. A large prospective observational study data (*n* = 229,230) suggests that acupuncture is associated with a very low risk of local infection in the general population (0.014%) treated with acupuncture [20]. There is also some preliminary evidence that acupuncture may shorten the duration of neutropenia and speed up recovery of the immune system during certain types of chemotherapy [37, 38], as well as being an effective intervention for some other treatment related side effects [39, 40].

There is a lack of evidence for a significantly increased infection risk from acupuncture given during treatment. Many major cancer centres in the USA, Australia, UK, Israel, Asia, and Europe offer acupuncture alongside or during immunosuppressive SACT. However, some oncologists work on the precautionary principle and prefer their patients to avoid acupuncture during the “nadir” period of chemotherapy cycles (when the white blood count is at its lowest — the timing of this varies with different chemotherapy regimens) or to avoid acupuncture completely until chemotherapy is completed. The potential risk of acupuncture is likely to be significantly less than that of taking blood or inserting an intravenous cannula, by virtue of the size and shape of the needles used.

Using clinically clean, minimal contact needling techniques and sterile, single-use needles ensures that acupuncturists keep any risk to a minimum.

#### Thrombocytopenia

Acupuncturists working in settings (e.g. haematology units) where patients are more likely to have severe thrombocytopenia (low platelet counts) should liaise with the primary oncology healthcare team to assess the individual’s clinical picture, especially in the case of complex or unstable cases.

To minimise bleeding in most patients, published expert opinions cite platelet counts of 20 × 10^9^/L (20,000/mm^3^) as a safe cut-off point [17, 41], whilst clinicians working in oncology settings may have locally agreed practices that differ slightly. Below this level acupuncturists should only use needles with the agreement of the treating oncology team and might consider using non-penetrative alternatives to needling and avoid firm pressure when applying acupressure.

In the community, patients who report spontaneous bleeding or bruising should be referred as per Red Flag guidance (Table 4). Patients without known thrombocytopenia and without symptoms or a history of bruising or bleeding are unlikely to have platelet counts below 20 × 10^9^/L (20,000/mm^3^).

#### Summary practice recommendations for treating patients with low blood counts

Patients may find they are given conflicting or confusing advice by different professionals. Co-ordination of care and good communication can help maintain safety and mitigate this situation. The acupuncturist should explain the theoretical risks and benefits to the patient who should ensure their oncologist is aware that acupuncture is being offered and has a chance to express any concerns.

- When current blood count data is available to the acupuncturist: for patients whose nadir neutrophil count does not drop below 0.9 × 10^9^/L (900/mm^3^) and whose platelets do not drop below 20 × 10^9^/L(20,000/mm^3^), acupuncture can be given safely throughout the chemotherapy cycle. Acupuncture should not be given whilst neutrophil counts are below 0.5 × 10^9^/L (500/mm^3^) or whilst platelet counts are below 20 × 10^9^/L (20,000/mm^3^).
- When blood count data is not available: in situations outside oncology centres, where blood count results are not available, acupuncture treatments should ideally be given on the day of, or within a few days after SACT administration, to avoid the nadir period which varies with treatment type and schedule. Patients can ask their oncology team when their nadir is likely to be.

For all practitioners, do not treat patients with the following conditions unless expressed consent has been given by the treating medical haematology/oncology team:

- Patients who are severely and ongoingly immuno-compromised (neutrophil count < 0.1 × 10^9^/L (100/mm^3^) because of ongoing immunosuppressive treatments (e.g. who are inpatients during or following bone-marrow/stem cell transplantation).
- Patients being treated intensively for blood or bone marrow cancers (leukaemias, myelomas, plasmacytomas, myelodysplastic syndromes, and some lymphomas) or having bone marrow transplantation, because treatments cause more profound and significant neutropenia.

#### Auto-immune reactions to immunotherapy

Acupuncturists should be aware that patients receiving immunotherapy, particularly immune checkpoint inhibitor therapy, may be susceptible to a wide range of auto-immune inflammatory responses that cause damage to the body’s organs (see Table 5) [42]. These side effects may be serious or even life-threatening. Any new symptoms suggestive of auto-immune problems should be urgently assessed as a Red Flag by the patient’s oncologist, an Acute Oncology Service (AOS), or the patient may need emergency medical care if they are systemically unwell.

**Table 5:** Possible side effects of immunotherapy, particularly checkpoint inhibitor therapies

Common	Less common
Digestive system • Colitis: stomach or abdominal pain, diarrhoea ± mucus, black or bloody stools Endocrine system — inflammation affecting: • Pituitary gland: headaches, fatigue • Adrenal gland: fatigue, low blood pressure (dizziness or fainting), muscle weakness, loss of appetite, weight loss, abdominal pain • Thyroid gland: hypothyroidism (weakness, constipation, dry skin, weight gain, sensitivity to cold); hyperthyroidism (anxiety, palpitations or other cardiac arrhythmias, diarrhoea, tremor, weight loss, sweating, sensitivity to heat) Pancreas • Pancreatitis (severe abdominal pain, nausea, vomiting, pale floating stools) Musculoskeletal system • Myositis: inflammation of the muscles leading to pain and weakness • Arthritis: inflammation, swelling, stiffness and joint pain Respiratory system • Pneumonitis: shortness of breath, bad cough Skin • Rashes, itchy skin, blisters, sores	Digestive system **• **Hepatitis: yellow skin and eyes, nausea or vomiting, stomach pain, dark urine, generalised itching, bleeding, or bruising Cardiovascular **• **Myocarditis: lowered blood pressure and in rare cases reduced ability to pump blood and disrupted heartbeat leading to heart attack Renal system **• **Nephritis: decreased urine production, blood in urine Nervous system **• **Encephalitis: mild flu-like symptoms, or more severe episodes include sudden and high fever, confusion, hallucinations, seizures, and vomiting

Given that immunotherapy is a relatively new form of anti-cancer treatment, the benefits of adjunctive acupuncture in these situations are still largely unproven, although anecdotal reports and clinical experience with non-immunotherapy-induced auto-immune disease point to potentially promising results. Animal studies of acupuncture report reduced inflammation and pain in rheumatoid arthritis [43], and it can be used in the adjunctive treatment of a range of auto-immune diseases [44]. Its potential to reduce this type of auto-immune reaction, and thereby increase tolerance and effectiveness of immunotherapy, warrants larger rigorous research studies.

### Hormonal therapies

Acupuncture is a safe and evidence-based intervention for treating the side effects of hormonal anti-cancer treatments, including aromatase inhibitor-related joint pain [7], hot flushes, sleep problems, anxiety [10], and distress [4]. Evidence for acupuncture for breast cancer treatment related hot flushes reports that acupuncture is acceptable to clinicians and to cancer survivors [45]. Acupuncture may be preferred by patients who do not wish to take further pharmaceutical products other than their adjuvant treatments, with research reporting acupuncture is as effective for hot flushes as gabapentin [46] and venlafaxine [47], with fewer side effects and less rebound effect after treatment ends. This also avoids any problems of interaction of pharmaceuticals with adjuvant hormonal therapies (e.g. interactions between tamoxifen and some anti-depressants). Prostate cancer survivors on hormone therapy may also benefit from acupuncture for managing hot flushes and related distress [48].

There is limited evidence that acupuncture alone causes any change in circulating hormone levels. (Acupuncturists who also use or recommend herbal treatments should be aware that some herbal preparations contain significant quantities of phyto-hormones and could potentially reduce the effectiveness of any hormone-blocking cancer treatment.)

### Radiotherapy

Acupuncture can be useful for managing side effects of radiotherapy, including radiation-induced xerostomia (RIX, dry mouth), radiation fatigue, and radiation-induced nausea and vomiting (a consequence of radiotherapy to the upper abdomen) [12, 49]. The following should be observed:

- Needles should not be inserted into an area that has received radiotherapy within the previous month, or where there is any residual redness, blistering, swelling, oedema, skin fragility, hypersensitivity, or broken skin because of radiation.
- For the safety of the acupuncturist, patients who have received radiopharmaceuticals as part of theranostics or other treatment plans should not be given acupuncture until their levels of radiation have returned to normal as advised by their oncology team (see Table 3).
- Lymphoedema can be a risk after radiotherapy to regional lymph nodes, even if surgical removal of lymph nodes has not been performed (see the “Lymphoedema and risk of lymphoedema” section).

## Conclusion

Acupuncture offers a non-pharmaceutical, evidence-based approach to managing many uncomfortable consequences of cancer and its treatments. It should be routinely considered, and ideally offered, as part of the multi-disciplinary approach to cancer care, as it can be administered safely within and outside of oncology settings, at any stage of the cancer survivorship pathway. These recommendations clarify the principles, knowledge, and practices which are necessary for the safe and appropriate administration of acupuncture to people affected by cancer. They aim to upskill and empower practitioners and protect patients. It is also hoped that the publication of these recommendations will encourage policy makers and healthcare providers to regard acupuncture as a safe and effective part of routine care for people living with and beyond cancer.

## Data Availability

Not applicable.
